# Impact of sleeve gastrectomy on bone metabolism in obese patients: from mechanisms to clinical prevention

**DOI:** 10.3389/fmed.2026.1728174

**Published:** 2026-02-18

**Authors:** Dawei Fan, Ce Fu, Baogang Zhu, Yan Wang, Pengfang Sheng, Jianghua Feng, Xiaopeng Wang

**Affiliations:** 1 The First Clinical Medical College of Gansu University of Chinese Medicine, Lanzhou, China; 2 Gansu Provincial People’s Hospital, Lanzhou, China

**Keywords:** bone metabolism, bone mineral density, bone turnover markers, obesity, sleeve gastrectomy, bone metabolism disorders, weight loss

## Abstract

In recent years, the global incidence of obesity has continued to rise, making obesity and its associated complications a serious threat to human health. Bariatric surgery, represented by sleeve gastrectomy (SG), has emerged as a crucial intervention for the treatment of obesity and related metabolic comorbidities. However, accumulating evidence indicates that bariatric surgery exerts multiple adverse effects on bone tissue, characterized by significantly increased bone resorption, decreased bone mineral density (BMD), and a consequent elevation in fracture risk. This review summarizes the effects of SG on BMD, bone turnover markers (BTMs), and bone microarchitecture in obese individuals, and comprehensively discusses the underlying mechanisms as well as corresponding clinical prevention strategies.

## 1 Introduction

Obesity has become one of the most prevalent chronic non-communicable diseases in the 21st century, characterized by a global prevalence, youth predominance, and increasing severity. Although lifestyle interventions (including dietary control and exercise therapy) remain the cornerstone of obesity management, they are often ineffective in patients with moderate-to-severe obesity (body mass index, BMI ≥ 35 kg/m^2^) or those with severe metabolic complications such as poorly controlled type 2 diabetes mellitus or refractory hypertension (1). Sleeve gastrectomy (SG) has been validated as a widely applicable bariatric procedure that effectively reduces body weight and improves obesity-related comorbidities (2). Nevertheless, emerging evidence suggests that bariatric surgery adversely affects bone metabolism, disrupting postoperative bone homeostasis, significantly enhancing bone resorption, and ultimately leading to decreased BMD, impaired bone strength and toughness, and a substantial increase in fracture risk. Potential mechanisms involve impaired nutrient absorption, reduced mechanical loading, hormonal alterations, gut microbiota dysbiosis, and abnormal accumulation of marrow adipose tissue (MAT) (3). Currently, systematic research on postoperative bone metabolic abnormalities following SG remains insufficient, particularly regarding long-term impacts on bone health and precise intervention strategies. Therefore, a comprehensive exploration of the mechanisms underlying SG-induced bone metabolic changes and the development of corresponding clinical preventive measures are of great clinical significance for reducing bone-related adverse events after surgery.

## 2 Dual effects of obesity on BMD and population heterogeneity

Obesity is generally associated with higher BMD. The underlying mechanisms mainly include continuous mechanical loading on the skeleton in the obese state, which stimulates osteoblast activity and promotes bone matrix synthesis and mineralization, as well as the aromatase-mediated conversion of androgens to estrogens in adipose tissue (especially visceral fat). Collectively, these factors exert a baseline protective effect on BMD, resulting in higher baseline BMD in obese individuals compared with normal-weight subjects (4–7). However, the impact of obesity on bone tissue exhibits a “double-edged sword” characteristic. Although moderate mechanical loading may stimulate bone formation, chronic overload can impair trabecular microarchitecture; even with normal BMD, bone fragility may still increase. When obesity is accompanied by metabolic abnormalities such as insulin resistance, inflammatory factors and advanced glycation end products can disrupt bone metabolism, offsetting the aforementioned protective effects (8–11). Notably, the impact of different obesity phenotypes on BMD varies: general obesity, as measured by BMI, shows a positive correlation with BMD, whereas abdominal obesity, as assessed by the a body shape index (ABSI), is negatively correlated with BMD in adolescents. This suggests that the metabolic toxicity of visceral fat may exert a more pronounced negative effect on bone health (12).

The influence of obesity on bone metabolism varies across populations: (1) Children and adolescents: While obesity may promote bone accumulation through increased mechanical loading, it can also induce bone biomechanical abnormalities (e.g., altered bone morphology and uneven stress distribution), thereby increasing the risk of long-bone fractures (e.g., femur and tibia) (13); (2) Postmenopausal women: Ovarian dysfunction and endogenous estrogen deficiency significantly weaken the protective effect of obesity on BMD, and leptin secreted by excessive adipose tissue may induce leptin resistance, further exacerbating bone loss (14); (3) Elderly individuals: The coexistence of obesity and sarcopenia, termed “sarcopenic obesity,” can significantly increase bone fragility and fracture risk by impairing the muscle-bone coupling mechanism (e.g., reduced mechanical stimulation from muscle to bone) (15); (4) Normal-weight obesity: Individuals with normal BMI but high body fat percentage exhibit significantly lower BMD than healthy controls, indicating that the “quality” of fat distribution (e.g., the proportion of visceral fat) rather than fat “quantity” alone plays a more critical role in regulating bone health (12). Thus, due to population heterogeneity, obese patients already present with varying baseline bone health status and metabolic vulnerability prior to surgery. This further suggests that after undergoing SG, bone metabolic responses and bone loss risk may also exhibit population specificity and be influenced by preoperative bone status.

## 3 Impact of SG on BMD

Numerous studies have demonstrated a persistent decline in BMD following SG. BMD at the lumbar spine, femoral neck, and total body is significantly reduced postoperatively, a trend that remains unresolved even 2 years after surgery (16). A study involving 33 obese female patients showed that lumbar spine BMD decreased by approximately 6.2% and femoral neck BMD by about 7.7% 2 years after SG (17). Even 5 years postoperatively, lumbar spine and femoral BMD remain significantly lower, with more pronounced bone loss observed in trabecular-rich regions (16). Compared with Roux-en-Y gastric bypass (RYGB), SG exerts a relatively milder impact on BMD. The reduction in lumbar spine BMD after RYGB (approximately 10%) is greater than that after SG (about 6–8%), and RYGB patients also exhibit a higher fracture risk (18, 19). Additionally, gender differences influence postoperative BMD changes, with females experiencing more significant BMD decline, which may be associated with fluctuations in estrogen levels after surgery (20).

Among bone density measurement techniques, dual-energy X-ray absorptiometry (DXA) is primarily used to assess bone mineral content (BMC) and areal bone mineral density (aBMD). In contrast, high-resolution peripheral quantitative computed tomography (HR-pQCT) can simultaneously measure volumetric bone mineral density (vBMD), bone geometry, and microarchitecture (21). Notably, in obese populations, DXA measurements are susceptible to interference from extra-osseous tissues and other artifacts, potentially leading to biased results. HR-pQCT not only accurately measures vBMD but also clearly reveals changes in bone microarchitecture, providing more detailed and precise detection outcomes (22). Further analyses using trabecular bone score (TBS) and 3D-DXA have shown that after SG, trabecular number decreases and trabecular separation increases, leading to deterioration of bone microarchitectural quality (23). The cross-sectional area and cortical thickness in trabecular-rich hip regions (e.g., intertrochanteric area) are also significantly reduced (19). Overall, SG exerts a more pronounced negative effect on trabecular bone (e.g., lumbar spine). Although cortical BMD in peripheral skeletal sites (e.g., distal radius and tibia) may transiently increase, overall bone strength is still compromised (24).

## 4 Imbalance of BTMs after SG

Multiple studies have shown that BTMs exhibit a characteristic imbalance after SG, specifically manifested by significant increases in both bone formation markers (e.g., procollagen type I N-terminal propeptide, P1NP) and bone resorption markers (e.g., C-terminal telopeptide of type I collagen, CTX). This indicates a state of high-turnover bone loss postoperatively (i.e., simultaneous acceleration of bone formation and resorption, with resorption predominating) (25). From a temporal dynamic perspective, 1 year after surgery, CTX and P1NP increase by approximately 100 and 50% from baseline, respectively (16). CTX rises rapidly after surgery, peaking within 1 year (about a 100% increase), and its elevation shows a significant positive correlation with BMD decline. Although P1NP also increases significantly, its magnitude is lower than that of bone resorption markers, further confirming the imbalance between bone formation and resorption (26). Two years postoperatively, bone resorption markers remain elevated, while the increase in bone formation markers slows slightly but remains significantly higher than preoperative baseline levels (17). Even 5 years after surgery, BTMs do not return to preoperative levels, suggesting that bone metabolic abnormalities after SG may be long-lasting (24). Additionally, changes in other BTMs reflect disturbances in bone metabolism: Tartrate-resistant acid phosphatase (TRACP)-5b continues to rise postoperatively, indicating enhanced osteoclast activity (15). Osteocalcin increases after surgery, which may be related to postoperative vitamin D deficiency or secondary hyperparathyroidism (SHPT). Vitamin D deficiency, SHPT, and increased urinary calcium excretion further exacerbate bone loss (27). Regarding population and procedural differences, adolescents show a greater increase in bone resorption markers after SG, with CTX rising by approximately 120%, possibly due to active bone metabolism and increased calcium demand during puberty (28). Compared with RYGB, SG has a relatively milder impact on BTMs; the increase in *β*-CTX after RYGB is about 1.5 times that after SG, but both are significantly higher than those in non-surgical controls (25).

## 5 Potential mechanisms underlying the impact of SG on bone metabolism

The negative impact of SG on bone metabolism is not attributable to a single independent factor but results from a cascade reaction in which impaired nutrient absorption and reduced mechanical loading act as core triggers. These factors interact with two major intermediary pathways—hormonal disruption and gut microbiota remodeling—and ultimately lead to abnormal accumulation of MAT, forming a closed causal loop that collectively disrupts bone metabolic balance. The specific cross-interactions among these factors are detailed below.

### 5.1 Impaired nutrient absorption

SG involves the resection of approximately 80% of the stomach (mainly the fundus and body), leading to anatomical and functional impairments in nutrient absorption, thereby interfering with bone metabolic balance. On one hand, the reduction in fundic and body tissue results in a marked decrease in gastric acid secretion. An acidic environment is crucial for calcium ionization; insufficient gastric acid directly reduces calcium absorption efficiency. On the other hand, the drastic reduction in gastric capacity (postoperative volume reduced to 100–150 mL) leads to inadequate food grinding and a decreased contact area between digestive enzymes and food, ultimately reducing the absorption of key bone metabolism-related nutrients such as calcium, magnesium, vitamin D, and protein. Long-term deficiency of these nutrients directly inhibits bone formation and increases the risk of osteoporosis. It also indirectly disrupts gut microbiota homeostasis, reduces short-chain fatty acid production, and diminishes the synthesis of bone-protective hormones such as growth hormone (GH) and estrogen, laying the foundation for subsequent hormonal disturbances and microbiota imbalance. Calcium and vitamin D deficiency can induce SHPT, leading to excessive parathyroid hormone secretion, which activates osteoclasts, accelerates bone resorption, and reduces bone mineralization efficiency, resulting in continuous bone loss (29). Deficiency of vitamin K2, which promotes calcium deposition in bone, can lead to bone calcium loss; magnesium deficiency inhibits osteoblast activity; and insufficient protein directly affects the synthesis of bone matrix (mainly composed of collagen), weakening skeletal structural support and further increasing the risk of osteoporosis (30).

### 5.2 Hormonal alterations

SG resects most of the gastric fundus, which is rich in endocrine cells, directly causing a significant decrease in ghrelin levels postoperatively, while intestinal hormones such as glucagon-like peptide-1 (GLP-1) and peptide YY (PYY) increase markedly. Although these hormonal changes contribute to weight loss by suppressing appetite and regulating glucose metabolism, the reduction in ghrelin indirectly affects GH secretion. As a key hormone that promotes osteoblast activity and maintains BMD, insufficient GH secretion impairs bone formation (31). Further confirmation in an obese rat SG model showed that, compared with sham-operated rats, SG rats exhibited significantly reduced insulin and ghrelin levels, while GLP-1 and adipokines (e.g., adiponectin) increased. GLP-1, a hormone closely related to bone metabolism, may indirectly participate in bone metabolic imbalance when abnormally elevated (32). Post-SG reduction in visceral fat alters adipokine secretion patterns: Adiponectin levels rise, which can inhibit osteoblast proliferation and differentiation, thereby weakening bone formation; leptin levels decrease, diminishing its bone-protective effects (e.g., promoting osteoblast activity). This dual effect exacerbates bone metabolic disturbance (33). Studies have found that gut microbiota metabolites such as short-chain fatty acids (SCFAs) can regulate the expression of sex hormone-binding globulin (SHBG), thereby influencing the bioavailability of sex hormones such as estrogen and testosterone, forming a “nutrition→microbiota→hormone” regulatory axis. Postoperative reductions in body weight, lean mass, and steroid hormone levels are positively correlated with bone loss. Increased SHBG levels in male patients after SG show a significant positive correlation with BMD decline, while female patients are less affected by SHBG changes, indicating gender-specific mechanisms in the hormonal regulation of bone metabolism (20). Moreover, sclerostin levels increase by approximately 20% after SG. By specifically inhibiting the Wnt/*β*-catenin signaling pathway, sclerostin directly reduces osteoblast generation and bone matrix synthesis, further aggravating bone loss (32). Collectively, these studies indicate that hormonal disruption interacts with impaired nutrient absorption and gut microbiota changes, and the multidimensional alterations in hormone levels after SG constitute an important mechanism mediating bone metabolic abnormalities.

### 5.3 Mechanical loading–bone stress regulation axis

After SG, patients lose an average of approximately 30% of their body weight, leading to a significant reduction in mechanical loading on the skeleton. This reduction in loading may directly weaken bone formation by inhibiting the Wnt/*β*-catenin signaling pathway. Reduced loading also indirectly affects the muscle-bone coupling mechanism, leading to decreased leptin secretion and further promoting MAT accumulation (28). Animal experiments have shown that osteocytes are particularly sensitive to biomechanical stress. Mechanical loading on bone is a crucial factor in maintaining BMD; under unloading conditions, osteocyte apoptosis increases, osteoclast activity is enhanced, and osteoblast differentiation is suppressed, resulting in decreased BMD. Conversely, appropriate mechanical loading can reduce osteocyte apoptosis, inhibit osteoclast function, promote osteoblast differentiation, and thereby maintain or even increase BMD. Furthermore, the substantial weight loss after SG leads to sustained reduction in skeletal stress, which activates the key bone resorption signaling pathway involving receptor activator of nuclear factor-κB ligand/osteoprotegerin (RANKL/OPG), ultimately accelerating bone loss (34).

### 5.4 Gut microbiota changes and bone metabolism

Alterations in gut microbiota may contribute to impaired bone metabolism. Post-SG changes in gut microbiota composition can indirectly affect bone metabolism by regulating SCFAs, bile acid metabolism, immune responses (e.g., regulating Th17/Treg balance), and inflammatory factors (e.g., IL-6, TNF-*α*) (35). *Clostridium butyricum*, as a probiotic, is beneficial to skeletal health. In a study by Shang et al., *C. butyricum* intervention alleviated bone density decline, reduced CTX levels, increased BMD, improved trabecular microarchitecture and bone histopathology, regulated osteoblast and osteoclast activity, increased bone collagen content, modulated RANKL and OPG expression, mitigated bone loss induced by gut microbiota changes after bariatric surgery, and promoted osteoblast autophagy (36). A prospective cohort study of 23 severely obese patients undergoing laparoscopic SG found a decreased relative abundance of Firmicutes and reduced Bifidobacterium in the gut microbiota. Greater postoperative changes in microbial community composition were associated with higher increases in P1NP and more significant femoral neck bone loss. Increased microbial diversity correlated with elevated IGF-1 levels; postoperative fecal butyrate concentration was positively correlated with IGF-1 levels and negatively correlated with CTX levels. These findings suggest that alterations in gut microbiota may be a factor contributing to adverse skeletal outcomes after laparoscopic SG, possibly related to reduced SCFA production affecting bone turnover and IGF-1 levels (37).

### 5.5 Abnormal accumulation of marrow adipose tissue

MAT acts as a key effector in bone metabolic abnormalities; its accumulation results from the synergistic action of all the aforementioned factors and, in turn, exacerbates bone loss. Hormonal disturbances directly promote the differentiation of bone marrow mesenchymal stem cells into adipocytes while inhibiting their differentiation into osteoblasts. Reduced mechanical loading leads to increased osteocyte apoptosis and expansion of the marrow cavity, providing “physical space” for MAT infiltration. Gut microbiota imbalance (reduced SCFAs) further accelerates fat accumulation in the marrow by downregulating IGF-1 levels. Increased vertebral MAT in adolescent obese patients after surgery correlates with BMD decline, suggesting that an imbalance between adipocyte differentiation and osteoblast activity may underlie reduced bone strength (38). One year after SG, lumbar MAT increases significantly, which may further weaken bone strength by inhibiting osteoblast activity. In contrast, MAT in peripheral bones (e.g., tibia) may decrease, possibly related to systemic metabolic changes (39). High MAT levels are significantly associated with low BMD and deteriorated bone microarchitecture, particularly in adolescent patients, indicating that MAT could serve as a novel marker for assessing postoperative bone health (40).

## 6 Clinical management strategies

### 6.1 Calcium and vitamin D supplementation and monitoring of BTMs and BMD

According to guidelines from the American Society for Metabolic and Bariatric Surgery (ASMBS) and the joint guidelines of the American Association of Clinical Endocrinologists/The Obesity Society/ASMBS (AACE/TOS/ASMBS), all patients undergoing bariatric surgery require lifelong nutritional monitoring and supplementation to mitigate postoperative bone loss and the risk of related metabolic bone diseases.

Vitamin D deficiency (serum 25(OH)D < 30 ng/mL) should be corrected preoperatively. Postoperatively, daily supplementation with 3,000–6,000 IU of vitamin D₃ is recommended, with individualized dose adjustment based on serum 25(OH)D levels (target > 30 ng/mL). For patients with persistent deficiency, short-term high-dose oral or intramuscular regimens may be considered. The target daily intake of elemental calcium is 1,200–1,500 mg, with calcium citrate preferred (its absorption is not affected by gastric acid secretion); divided doses are advised to enhance absorption efficiency (41).

Monitoring regimen: During the first postoperative year, serum 25(OH)D, calcium, phosphorus, and parathyroid hormone (PTH) should be measured every 3–6 months; once stable, annual monitoring is sufficient (42). BTMs monitoring: Assessment of bone resorption markers (e.g., CTX) and bone formation markers (e.g., P1NP) is recommended preoperatively, 6 months postoperatively, and annually thereafter to dynamically evaluate bone turnover status. Persistently elevated CTX suggests active bone loss and warrants intensified intervention. BMD monitoring: All patients should undergo baseline DXA examination 1–2 years after surgery, focusing on the lumbar spine and hip. If no significant bone loss is observed, DXA can be repeated every 2 years. If BMD declines markedly (T-score < −2.0 or annual decrease > 5%), the monitoring interval should be shortened to annually, and further evaluation (e.g., HR-pQCT or TBS) may be considered (42).

### 6.2 Advances in pharmacological therapy

Anti-resorptive agents: Bisphosphonates have shown certain bone-protective effects in SG patients, primarily by inhibiting osteoclast activity to slow bone loss, but their long-term efficacy and safety require further verification (43). Bone-forming agents: The potential of parathyroid hormone analogs (e.g., teriparatide) is being evaluated; however, their widespread application is limited by injectable administration and high treatment costs (44). Gut microbiota modulation: As an emerging research direction, post-SG gut microbiota changes are closely related to bone metabolism, suggesting that probiotic, prebiotic, or bile acid-based interventions may become future preventive strategies (45). GLP-1 receptor agonists: Agents such as semaglutide may exert dual effects on bone metabolism by modulating bone turnover balance while promoting weight loss, but their skeletal effects require further clarification (46).

Current pharmacological treatments still face challenges, including significant individual variability in efficacy, insufficient long-term safety data, and high costs. More randomized controlled trials are needed to clarify the effectiveness and safety of different drugs in the post-SG population.

### 6.3 Exercise and lifestyle interventions

Patients are advised to gradually engage in moderate-intensity weight-bearing exercise (e.g., brisk walking, stair climbing, resistance training) postoperatively, accumulating ≥150 min per week to maintain mechanical stimulation of the bone. Muscle-strengthening training: For individuals at risk of sarcopenic obesity, resistance training (e.g., elastic band exercises, weight training) should be combined, 2–3 times per week, to preserve muscle mass and provide mechanical support for bone health (42). Comprehensive management: Studies have confirmed that a combined intervention of vitamin D supplementation, a high-protein diet (≥60 g/day), and structured exercise yields significantly greater improvements in BMD and reduction in sclerostin levels compared with routine care (47). Implementing such comprehensive interventions is particularly important for young individuals undergoing bariatric surgery to mitigate potential adverse effects on skeletal health.

## 7 Perspectives

Post-SG decline in BMD results from the combined action of multiple factors, primarily including reduced mechanical loading, hormonal disturbances, gut microbiota changes, impaired nutrient absorption, and abnormal MAT accumulation. Although SG has a relatively milder negative impact on bone health compared with RYGB, enhanced monitoring of bone metabolism and targeted interventions (e.g., calcium/vitamin D supplementation, pharmacological therapy) remain necessary for adolescents, women, and long-term postoperative patients. Although the precise mechanisms by which SG affects bone health are not fully elucidated, systematic preoperative assessment and continuous postoperative management can partially alleviate the adverse skeletal effects of surgery. Notably, most existing studies have follow-up periods ≤2 years; long-term (≥5 years) bone metabolic changes and fracture risk after surgery remain unclear. Future research should focus on in-depth exploration of the interactions among postoperative gut microbiota, MAT, and bone metabolism, aiming to develop more precise prevention and treatment strategies to effectively safeguard long-term bone health in patients.
